# MiR−33 as a novel diagnostic biomarker for distinguishing cholesterol from adenomatous polyps: a case-control study

**DOI:** 10.1186/s41065-025-00407-6

**Published:** 2025-03-14

**Authors:** Xia Hu, Ping Zhang, Tong Wang, Quanzhi Li, Minjia Li, Zhuohan Zhao, Rui Yu, Yan Tan, Chengli Yao

**Affiliations:** 1 The First Clinical Medical College, Beijing University of Chinese Medicine, No. 5 Haiyuncang, Dongcheng District, Beijing, 100700 China; 2 The Second Department of General Surgery, Dongzhimen Hospital, Beijing University of Chinese Medicine, No.5 Haiyuncang, Dongcheng District, Beijing, 100700 China; 3 School of Life Sciences, Beijing University of Chinese Medicine, No.11 East Beisanhuan Road, Chaoyang District, Beijing, 100105 China

**Keywords:** Adenomatous polyp, Biomarker, Cholesterol polyp, MiR−33a, qRT-PCR, Statistical model

## Abstract

Cholecystectomy is often excessively utilized in the management of gallbladder polyps. It is crucial to effectively differentiate between adenomatous and cholesterol polyps to reduce unnecessary cholecystectomies. This study aimed to investigate the potential of miR−33 as a novel diagnostic biomarker for distinguishing cholesterol from adenomatous polyps. Gallbladder specimens were retrospectively collected from gallbladder polyp patients who underwent laparoscopic cholecystectomy at the Second Department of General Surgery, Dongzhimen Hospital, Beijing University of Traditional Chinese Medicine, between June 2021 and December 2021. Pathological analysis categorized the specimens into two groups: the cholesterol polyp group (*n* = 13) and the adenomatous polyp group (*n* = 12). The expression levels of miR−33a and miR−33b in both groups were assessed using real-time quantitative reverse transcription polymerase chain reaction (qRT-PCR). MiR-33a level and the miR-33a/miR-33b ratio were significantly lower in cholesterol polyps than in adenomatous polyps (*p* < 0.05). Spearman correlation analysis showed a strong positive correlation between miR-33a and miR-33b (*r* = 0.956, *p* < 0.001). Stepwise logistic regression analysis revealed that decreased miR-33b and elevated miR-33a/miR-33b ratio are independent risk factors for cholesterol polyps (*p* < 0.05). A predictive model was constructed, with the model’s AUC for diagnosing adenomatous polyps being 0.885 (95% CI: 0.753−1.000, *p* = 0.001), exhibiting a notable specificity of 84.62% and a sensitivity of 83.33% at a cut-off of 0.424. MiR−33 could serve as a novel diagnostic biomarker for distinguishing cholesterol from adenomatous polyps to facilitate the diagnosis and treatment of clinicians.

## Introduction

Gallbladder polyps (GBPs) are a group of various abnormally proliferated tissues that protrude into the gallbladder lumen. The global incidence of GBPs ranges from 0.9 to 12.1% [1], and the incidence varies among different populations. Pathologically, GBPs include cholesterol polyps (CP), gallbladder adenomyosis, inflammatory polyps, adenomatous polyps (AP) with malignant potential, and malignant gallbladder cancers [2]. Due to the lack of specific clinical manifestations, it is relatively difficult to accurately identify the types of gallbladder polyps. Gallbladder polyps with a diameter of ≥ 10 mm are frequently considered to have potentially malignant risk or indicate adenomas with malignant potential. This often leads clinicians to pursue aggressive therapeutic interventions, such as surgical excision, and even chemotherapy, immunotherapies and targeted treatments [3–5]. However, studies have shown that 43.6% (910 out of 2,085 cases in the study) to 99.6% (2,047 out of 2,055 cases in the study) of the gallbladder polyps removed during cholecystectomy are pathologically diagnosed as non-neoplastic polyps [6–8]. This means that the current surgical criteria may lead to unnecessary cholecystectomies [8–11]. The removal of the gallbladder can cause dyspeptic symptoms such as duodenalgastric reflux, steatorrhea, and abdominal distension, and it can also increase the risk of colorectal cancer by 2.1 times [12–17]. Therefore, it is of great significance to accurately assess non-neoplastic and benign neoplastic polyps.

Cholesterol polyps constitute approximately 50 − 70% of GBPs cases, while adenomatous polyps are the main type of neoplastic polyps. Effectively distinguishing between these two types of polyps can reduce unnecessary cholecystectomy procedures. Research shows that the formation of cholesterol polyps is closely related to disorders of lipid metabolism. Anna Stromsten et al. [18] discovered through biochemical analysis of the local mucosal components of gallbladder cholesterol polyps that the content of cholesterol esters in the tissue was 12 times higher than that of the control group. S Sahlin et al. [19] demonstrated that the cholesterol ester content in the gallbladder mucosa of patients with cholesterol deposition disease is positively correlated with the supersaturation of cholesterol in bile. The abnormal accumulation of cholesterol is an important feature of cholesterol polyps. When the reverse cholesterol transport (RCT) process is impaired, it leads to increased cholesterol deposition beneath the epithelium of the gallbladder mucosa, thereby promoting the formation of cholesterol polyps. MicroRNAs (miRNAs) are involved in numerous functions and processes in the body through the regulation of gene and protein expression. Dysregulation of miRNAs is associated with the development of several diseases, such as atherosclerosis [20], lipid metabolism disorders [21], brain cancer [22], and colon cancer [23]. Among these, MiR−33 is an important regulator in lipid metabolism. It can regulate cellular cholesterol levels by modulating the process of reverse cholesterol transport (RCT) [24–26]. Recent studies have further elucidated that miR-33a/b directly targets genes involved in fatty acid oxidation and HDL biogenesis, thereby influencing systemic lipid homeostasis [27, 28].

Based on the above-mentioned background, this study aims to explore the potential of miR−33a as a novel diagnostic biomarker for distinguishing between cholesterol polyps and adenomatous polyps by assessing miR−33 expression levels in the gallbladder mucosa.

## Materials and methods

### Study design

A retrospective case-control study, was conducted. Patients with gallbladder adenomas or cholesterol polyps who underwent laparoscopic cholecystectomy at the Second Department of General Surgery, Dongzhimen Hospital, Beijing University of Traditional Chinese Medicine, from June 2021 to December 2021, were enrolled.

The inclusion criteria were as follows: (a) cholecystectomy with postoperative pathological diagnosis of gallbladder cholesterol polyps or gallbladder adenoma, and (b) complete clinical baseline data. Exclusion criteria included: (a) acute cholecystitis and/or acute suppurative lesions, (b) gallstones and/or hepatic bile duct stones, (c) malignant gallbladder tumors. All procedures adhered to the ethical standards of the responsible committee on human experimentation (institutional and national) and to the Helsinki Declaration of 1975, as revised in 2008 [5]. This study was approved by the Ethics Review Committee of Dongzhimen Hospital Affiliated with Beijing University of Chinese Medicine (2022DZMEC−052−02). Due to the retrospective nature of the study, the Ethics Review Committee waived informed consent.

### Clinical data and miR-33 assay

The patients’ demographic information, including age, gender, body mass index (BMI), duration and size of the polyps, presence of hypertsion, hyperlipidemia, diabetes mellitus, fatty liver disease, and coronary heart disease, was gathered during enrollment.

The levels of miR−33a and miR−33b n the gallbladder mucosa were evaluated through real-time quantitative reverse transcription polymerase chain reaction (qRT-PCR). The standard protocol for qRT-PCR was referenced from previous studies [29, 30], and the specific steps are as follows:

a) Total RNA was extracted from the paraffin-embedded gallbladder tissue samples, and the concentration and purity of the RNA were determined by the spectrophotometric method.

b) Reverse Transcription: RNA was reverse-transcribed into cDNA using reverse transcriptase enzyme and the following primers:

miR−33a RT primer sequence: 5’ GTCGTATCCAGTGCGTGTCGTGGAGTCGGCAATTGCACTGGATACGACTGCAAT 3’.

miR−33b RT primer sequence: 5’ GTCGTATCCAGTGCGTGTCGTGGAGTCGGCAATTGCACTGGATACGACGCAATG 3’.

U6 RT primer sequence: 5’ CGCTTCACGAATTTGCGTGTCAT 3’.

The reverse transcription reaction was carried out under the following conditions: 25 °C for 5 min, 50 °C for 15 min, and 85 °C for 5 min. The synthesized cDNA was subsequently used for real-time fluorescence quantitative PCR without delay.

c) Primer Design and Preparation:

Primers for the target genes (miR−33a and miR−33b) and the reference gene (U6) were designed and prepared. The primer sequences used in the PCR reaction were as follows:

miR−33a primer sequence: 5’ GGCCGTGCATTGTAGTTGC 3’.

miR−33b primer sequence: 5’ GGCGTGCATTGCTGTTGC 3’.

U6 primer sequence: 5’ GCTTCGGCAGCACATATACTAAAAT 3’.

d) qPCR Reaction Setup:

The qPCR reaction was performed using cDNA, primers, and a pre-mixed qPCR master mix under optimized cycling conditions. The PCR reaction conditions were as follows: an initial denaturation at 95 °C for 30 s, followed by 40 cycles of denaturation at 95 °C for 10 s and annealing/extension at 60 °C for 30 s.

e) Data Analysis:

The specificity of the products was verified using a melting curve analysis, ensuring a single peak. The cycle threshold (Ct) values, representing the number of cycles required for the fluorescence signal to reach the set threshold, were recorded. The data were analyzed using the formula RQ = 2^-ΔΔCt method for relative quantification of gene expression levels, with U6 as an internal reference. ΔCt was computed as [Ct(miR-33) − Ct(U6)], and ΔΔCt was calculated as [ΔCt(adenomatous) − ΔCt(cholesterolous)]. The value of 2^-ΔΔCt represented the fold difference in miR-33 expression between adenomatous polyps and cholesterol polyps.

### Statistical analysis

Statistical analyses were conducted using SPSS 25.0. Normally distributed continuous variables were presented as mean ± standard deviation (X ± SD), while non-normally distributed continuous variables were expressed as median with interquartile range (IQR). Group comparisons were performed using Student’s t-test or the nonparametric Mann-Whitney U test, depending on the distribution of the variables. The Chi-square test was used for comparing categorical variables, presented as numbers (percentages). A two-tailed *p*-value < 0.05 was considered statistically significant.

Performed univariate logistic regression analysis and conducted collinearity diagnosis on the data, followed by multivariate logistic regression analysis (stepwise regression: backward, conditional) to identify independent risk factors. Utilized R language software version 4.2.0 to construct a nomogram for the regression prediction model. Employed Receiver Operating Characteristic (ROC) curve analysis to evaluate the effectiveness of the prediction model. Assessed the accuracy of the prediction model using calibration curve, decision curve analysis (DCA), and clinical impact curve. Applied the bootstrap method for internal validation of the model, which involved resampling the dataset with replacement to assess the stability and performance of the model.

## Result

### Comparison between groups

**Fig. 1:**
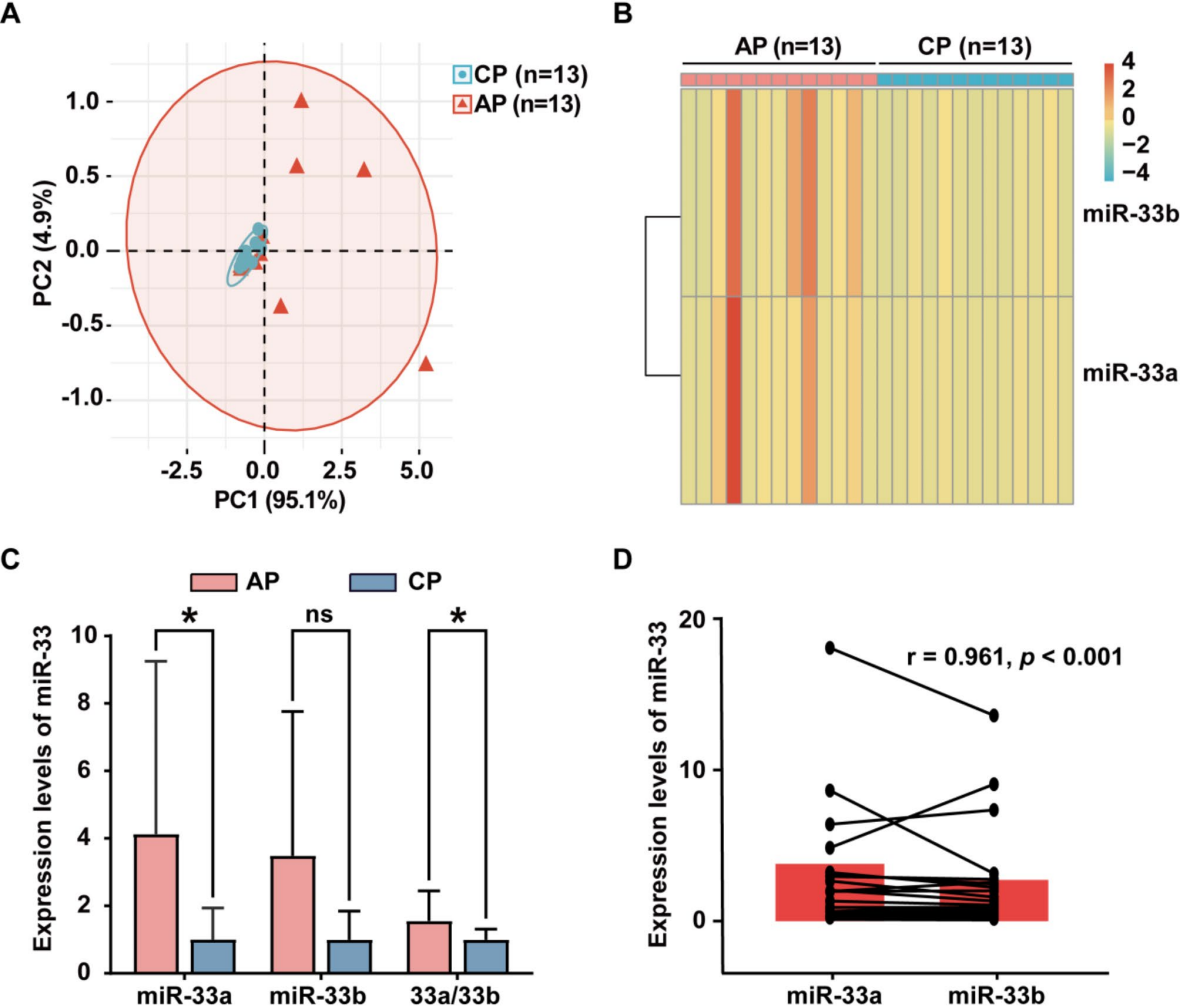
Comprehensive Analysis of miR-33 Expression. (**A**) Principal Component Analysis (PCA) of miR-33a and miR-33b expression in CP and AP. (**B**) Heatmap illustrating miR-33a and miR-33b expression in CP and AP. (**C**) Comparative analysis of miR-33a levels, miR-33b levels, and the miR-33a/miR-33b ratio between CP and AP groups. (**D**) Spearman correlation analysis of miR-33a and miR-33b expression in gallbladder polyps

According to the predefined inclusion and exclusion criteria, a total of 30 patients were initially enrolled in this study. However, 4 patients were subsequently excluded from further qRT-PCR testing during the RNA extraction phase due to inadequate RNA concentrations. We conducted principal component analysis (PCA) and heatmap visualization on the expression levels of miR-33a and miR-33b. The analysis revealed the presence of an outlier in the AP group, as depicted in Fig. 1A-B. Consequently, the final study cohort consisted of 25 patients, encompassing 13 cases of cholesterol polyps and 12 cases of adenomatous polyps (including 5 cases presenting both adenomatous and cholesterol polyps). The cohort comprised 10 males and 15 females, with a mean age of 52.96 ± 13.53 years.

A comparison between the groups revealed that both the miR-33a level and the miR-33a/miR-33b ratio were significantly lower in the cholesterol polyp group compared to the adenomatous polyp group (Table 1; Fig. 1C). Conversely, regarding known risk factors for gallbladder polyps such as age gender, MI, polyp duration and polyp size [31–34], as well as other general information including hypertension, hyperlipidemia, diabetes mellitus, fatty liver disease, coronary heart disease and the miR-33b level, there were no significant differences between the two groups (Table 1). Through Spearman correlation analysis, a strong positive correlation between miR-33a and miR-33b (*r* = 0.956, *p* < 0.001) (Fig. 1D).

**Table 1:** Comparative analysis of baseline characteristics and miR-33 levels across groups

	CP (*n* = 13)	AP (*n* = 12)	Value	*p*
Age (years)	48.00 ± 14.85	58.33 ± 9.91	t = 2.208	0.054
Gender (%)				
Male	5 (38.46%)	5 (41.67%)	χ2 = 0.027	0.870
Female	8 (61.54%)	7 (58.33%)		
BMI (kg/m2)	26.07 ± 2.32	25.51 ± 3.91	t = -0.428	0.674
Polyp duration (years)	0.25 ± 3.47	0.63 ± 5.58	Z = 0.901	0.376
Polyp size (cm)	0.90 ± 0.25	1.13 ± 0.56	t = 1.684	0.116
Hypertension	3 (23.08%)	5 (41.67%)	χ2 = 0.991	0.319
Hyperlipidemia	1 (7.69%)	1 (8.33%)	χ2 = 1.128	0.288
Diabetes mellitus	1 (7.69%)	1 (8.33%)	χ2 = 1.128	0.288
Fatty liver disease	1 (7.69%)	4 (33.33%)	χ2 = 2.564	0.109
Coronary heart disease	0 (0.00%)	1 (8.33%)	χ2 = 1.128	0.288
MiR-33a	0.58 ± 1.64	2.79 ± 5.55	Z = 2.013	0.046
MiR-33b	0.81 ± 1.32	1.89 ± 5.96	Z = 1.469	0.152
MiR-33a/miR-33b	1.00 ± 0.31	1.31 ± 0.69	Z = 2.013	0.046

### Regression analysis

Univariate analysis identified several risk factors associated with cholesterol polyps, such as younger age, reduced levels of miR-33a, and an elevated miR-33a/miR-33b ratio (Table 2). All *p*-values were below 0.1. Upon conducting collinearity diagnosis among the above risk factors and BMI, polyp duration, polyp size, miR-33b levels, a substantial collinearity was observed between miR-33a and miR-33b (5 < VIF < 10). Subsequent inclusion of these variables in a multivariate logistic regression analysis (stepwise, backward, conditional) highlighted decreased miR-33b levels and an increased miR-33a/miR-33b ratio as independent risk factors for cholesterol polyps (Table 3).

**Table 2:** Results of univariate logistic regression analysis

Variables	B	SE	*p*	OR	95% CI
Age	0.069	0.038	0.070	1.072	0.994, 1.155
BMI	-0.060	0.132	0.652	0.942	0.728, 1.220
Polyp duration	0.049	0.111	0.657	1.051	0.845, 1.307
Polyp size	2.141	1.451	0.140	8.507	0.495,146.233
MiR-33a	0.606	0.334	0.070	1.833	0.952, 3.528
MiR-33b	0.490	0.334	0.142	1.632	0.849, 3.138
MiR-33a/miR33b	2.178	1.271	0.087	8.829	0.731, 106.694

**Table 3:** Results of multivariate logistic regression analysis

Variables	B	SE	*p*	OR	95% CI
MiR-33b	0.727	0.367	0.048	2.068	1.007, 4.248
MiR-33a/miR33b	4.024	2.039	0.048	55.951	1.028, 3045.789
Intercept	-6.148	2.754	0.026	0.002	/

**Fig. 2:**
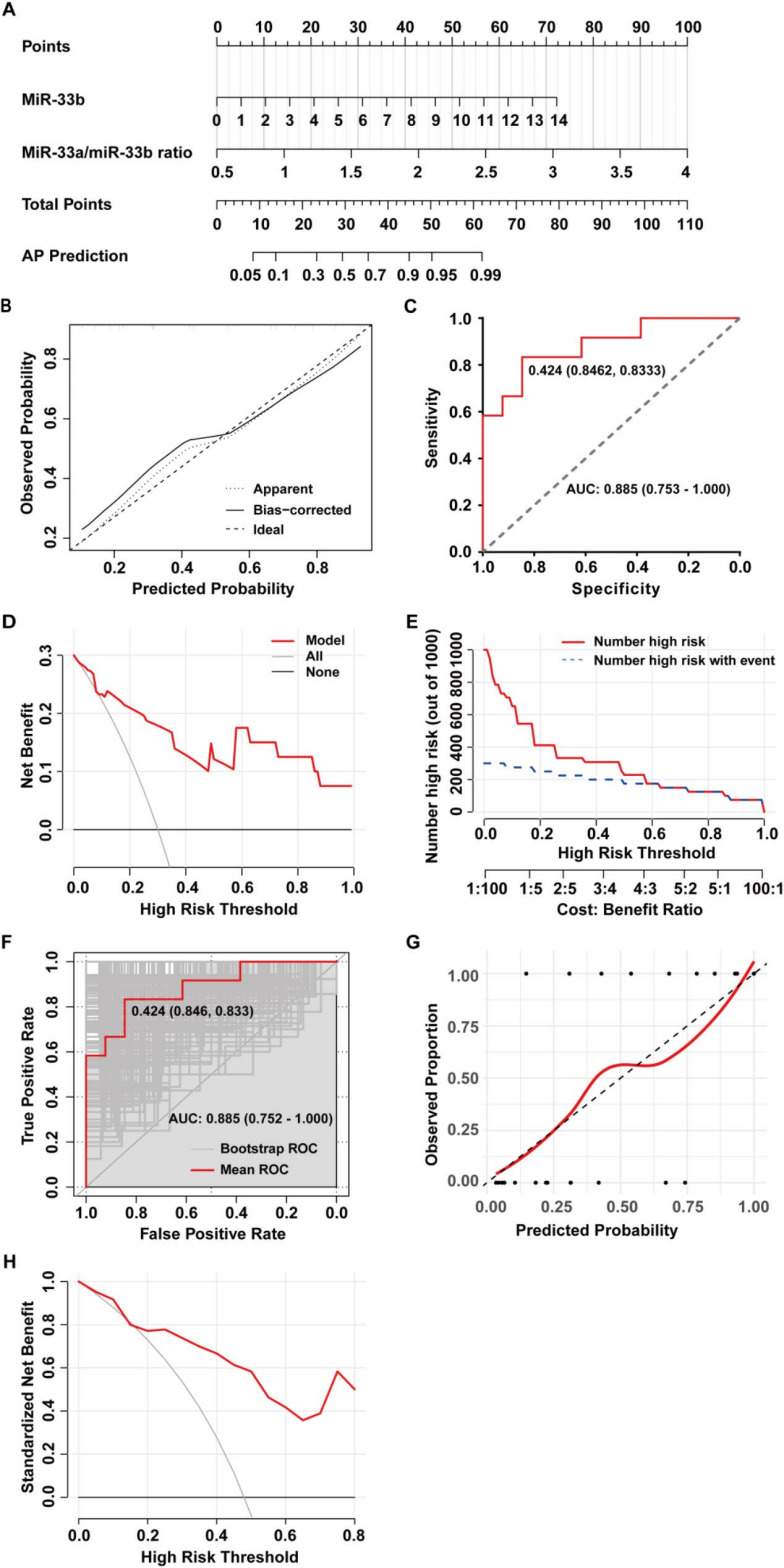
Performance Evaluation and Internal Validation of the Logistic Regression Analysis Model. (**A**) Nomogram of the logistic regression analysis model. (**B**) Correction curve of the logistic regression model. (**C**) ROC curve of the logistic regression analysis model. (**D**) Decision curve analysis of the logistic regression analysis model. (**E**) Clinical impact curve of the logistic regression analysis model. (**F**) ROC curve of internal validation using the bootstrap method. (**G**) Correction curve of internal validation using the bootstrap method. (**H**) Decision curve analysis of internal validation using the bootstrap method

A predictive model for distinguishing adenomatous polyps from cholesterol polyps was developed based on the outcomes of the multivariate logistic regression analysis. This model integrated two independent risk factors: decreased miR-33b levels and an elevated miR-33a/miR-33b ratio. The logistic regression equation was defined as follows: Logistic (P) = -6.148 + 4.024 * miR-33a/miR-33b ratio + 0.727 * miR-33b. Subsequently, a nomogram was created utilizing this predictive model (Fig. 2A). Through the calibration curve for assessing the model’s calibration, as shown in Fig. 2B, the calibration curve was very close to the 45-degree diagonal line, indicating the model’s excellent predictive ability to accurately predict the probability of the target event occurring. The area under the receiver operating characteristic (ROC) curve was calculated as 0.885 (95% CI: 0.753-1.000, *p* = 0.001) (Fig. 2C), demonstrating the model’s strong discriminatory power. The optimal cutoff value was 0.424, with a specificity of 84.62% and a sensitivity of 83.33%. The Decision Curve Analysis (DCA) showed a significant net benefit of using the AP nomogram model compared to the “all treatment” and “no treatment” strategies when the probability threshold was above 0.4 (Fig. 2D), confirming the model’s good discriminative ability within a relatively safe threshold range and validating its practicality in clinical applications. In the clinical impact curve (Fig. 2E), thresholds above 0.4 highlighted a high degree of alignment between model predictions and actual outcomes, emphasizing the model’s outstanding clinical predictive effectiveness.

Through internal validation using bootstrapping, the mean ROC curve showed an AUC of 0.885 (95% CI: 0.752-1.000, *p* = 0.001), a specificity of 67.27%, a sensitivity of 77.63%, and an accuracy of 71.21% (Fig. 2F). The internal validation calibration curve demonstrated that the scatter points predominantly align along a 45° diagonal line, indicating a strong consistency between the model’s predicted probabilities of event occurrence and the observed probabilities (Fig. 2G). The DCA resulting from internal validation indicated good discriminative ability when the probability threshold is above 0.5 (Fig. 2H). These results further confirmed the reliability and stability of the model, enhancing its credibility for practical clinical applications.

## Discussion

With the growing prevalence of community-based physical examinations, the incidence of gallbladder polyps (GBPs) has been progressively rising. Nevertheless, distinguishing between non-neoplastic and neoplastic polyps via imaging is a significant challenge. Given evidence that the majority of excised gallbladder polyps are benign [6–8, 10, 11], current management protocols are under review. Vigorous management of benign gallbladder polyps can lead harm for patients, leading to unnecessary surgeries, postoperative complications, frequent and extended follow-up imaging of uncertain advantage and patient distress and inconvenience [9–11, 35, 36]. It is imperative to accurately differentiate the subtypes of gallbladder polyps to streamline the diagnosis and treatment processes for healthcare providers.

MicroRNAs (miRNAs) are pivotal regulators of biological metabolism, exerting their effects through the negative regulation of target gene expression. Their high reliability and stability as well as accessibility in blood have made them increasingly recognized as promising biomarkers for disease diagnosis [37–44]. Among these, miR-33a is located within the intronic region of the Sterol Regulatory Element-Binding Protein-2 (SREBP-2) gene and is co-expressed with SREBP-2 [45–47]. SREBP-2 is a key transcription factor that regulates cholesterol synthesis and uptake. When intracellular cholesterol levels rise, the SREBP-2 pathway is activated, leading to the upregulation of miR-33a expression. However, elevated cholesterol levels also inhibit SREBP-2 activity through a negative feedback mechanism. Specifically, high cholesterol levels prevent the binding of sterol regulatory element-binding protein cleavage-activating protein (SCAP) to SREBP-2, blocking the effective transport of SREBP-2 to the endoplasmic reticulum and reducing its activity [45–48]. This feedback inhibition mechanism is crucial for maintaining cholesterol homeostasis, as it decreases cholesterol synthesis and uptake, preventing cellular damage from excess cholesterol.Concurrently, the reduction in SREBP-2 expression leads to decreased miR-33a levels [45, 49]. The down - regulation of miR-33a promotes the expression of cholesterol transporters such as ABCA1 and ABCG1, enhancing cholesterol efflux from cells and the production of high-density lipoprotein (HDL), thereby lowering intracellular cholesterol levels [45, 46].

JaiHoon Yoon et al. [50] discovered through Western blot and immunohistochemistry that the level of ABCA1 in the gallbladder of patients with cholesterol polyps was higher than that in normal gallbladders, and it was surrounded by cholesterol-rich macrophages. This indicates that ABCA1 plays a significant role in the reverse cholesterol transport and cholesterol efflux of gallbladder epithelial cells. Since miR-33 can inhibit the expression of ABCA1 [46, 48, 51], these findings imply a potential link between miR-33 and the pathogenesis of cholesterol polyps. However, prior to this study, no research had explored this association.

**Fig. 3:**
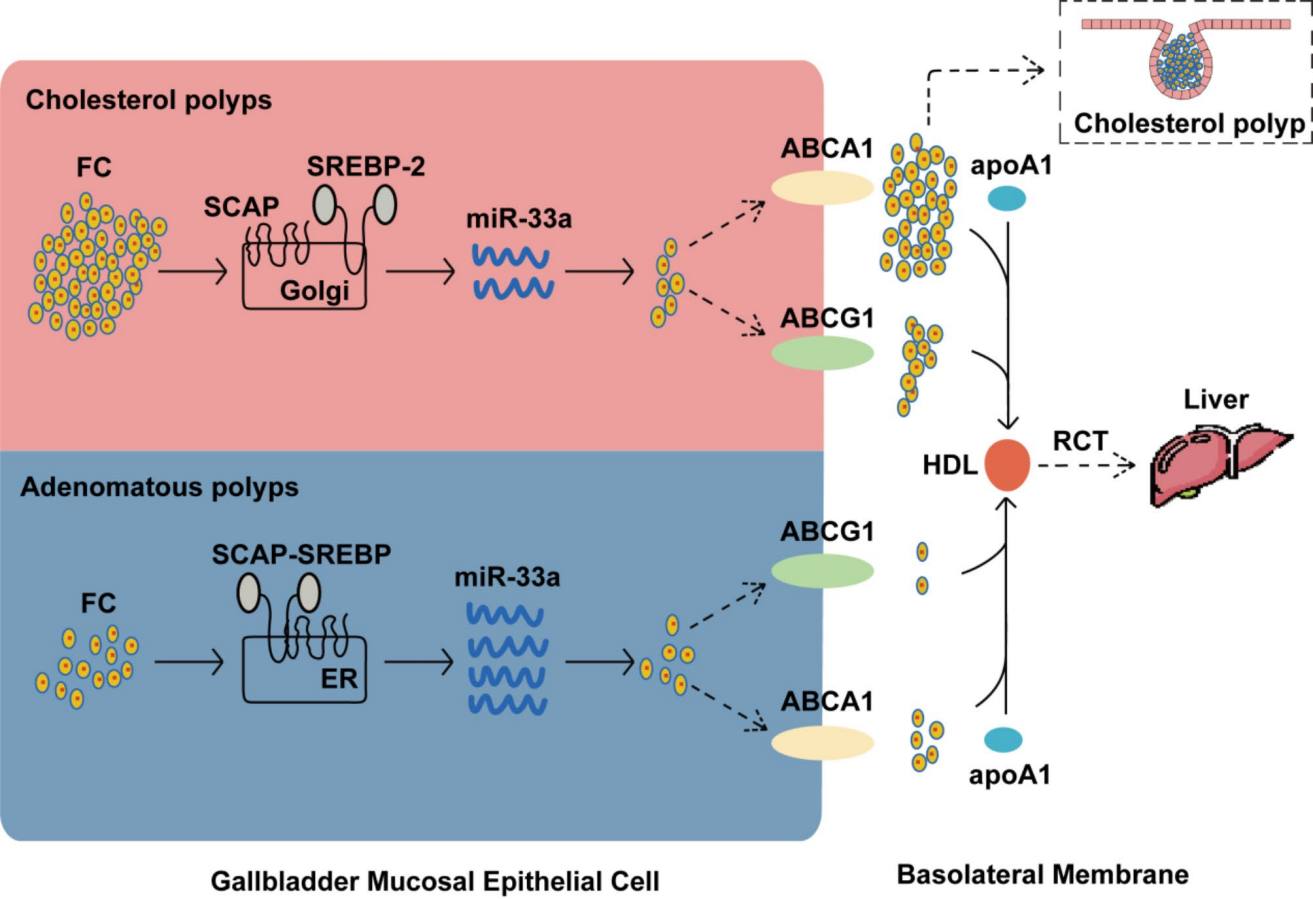
MiR-33a participates in the cholesterol efflux process in gallbladder mucosal epithelial cells of patients with cholesterol polyps

The study showed that, compared to the adenomatous polyp group, the cholesterol polyp group had significantly lower levels of miR-33a and a decreased miR-33a/miR-33b ratio compared to the adenomatous polyp group. Subsequent multivariate logistic regression analysis identified reduced miR-33b levels and an elevated miR-33a/miR-33b ratio as independent risk factors for cholesterol polyps. These findings support our hypothesis that miR-33a plays a role in the formation and development of cholesterol polyps. The underlying mechanism may involve the following: as intracellular cholesterol levels rise, miR-33a expression is downregulated through the negative feedback regulatory mechanism. This downregulation subsequently promotes the expression of ABCA1 and ABCG1, enhancing cholesterol efflux. The increased cholesterol efflux exacerbates lipid deposition beneath the gallbladder mucosa, thereby promoting cholesterol polyp formation (Fig. 3). Additionally, we observed a significant positive correlation between miR-33a and miR-33b expression in the gallbladder mucosa of polyp patients, suggesting coordinated regulation of these miRNAs in gallbladder pathologies.

Low expression of miR-33 in the gallbladder mucosa of patients with cholesterol polyps may be related to factors such as diet, lipid profile and genetic predisposition. Previous studies have shown that diet is involved in the regulation of miR-33 expression. A high-fat and high-cholesterol diet bidirectionally regulates the expression of miR-33 by activating the sterol regulatory element-binding protein-2 (SREBP-2) pathway and the cholesterol feedback regulation mechanism. When the cholesterol content in the diet is excessively high, the intracellular cholesterol level rises, activating the SREBP-2 pathway, and subsequently upregulating the expression of miR-33a. Meanwhile, an overly high intracellular cholesterol level can also inhibit the activity of SREBP-2 through a negative feedback mechanism, thereby reducing the expression of miR-33a [45–47, 51]. This dynamic equilibrium mechanism contributes to maintaining the homeostasis of intracellular cholesterol. In addition, a high-cholesterol diet may trigger inflammatory responses and oxidative stress, and these processes can also affect the expression of miR-33. For example, inflammatory factors (such as TNF-α and IL-6) may regulate the level of miR-33 through signaling pathways such as NF-κB [52].

Lipid profiles, including cholesterol, triglycerides, and lipoprotein levels, are closely associated with miR-33 expression. In individuals with abnormal lipid profiles, such as hypercholesterolemia or hypertriglyceridemia, miR-33 expression may be dysregulated. For instance, in patients with carotid atherosclerosis and low LDL levels, both miR-33a-5p and miR-33a-3p expression levels were significantly reduced and positively correlated with low-density lipoprotein cholesterol (LDL-C), suggesting a link between miR-33a and lipid profiles [41]. Additionally, Study [53] revealed that serum miR-33a expression levels were significantly elevated in patients with metabolic syndrome and showed a moderate positive correlation with lipid profile parameters, including triglycerides (TG), high-density lipoprotein (HDL), and low-density lipoprotein (LDL). These findings highlight the potential role of miR-33 in regulating lipid metabolism and its association with lipid-related disorders.

In addition, genetic factors also play a role in the expression of miR-33: Single nucleotide polymorphisms (SNPs) in the miR-33 gene or its regulatory regions can affect its transcription, processing, or stability [54]; Epigenetic factors such as DNA methylation can regulate the expression of miR-33b [55]; Genetic variations in miRNA biogenesis genes, such as Dicer or Drosha, may also disrupt the processing of miR-33.

In this study, we constructed a predictive model to effectively distinguish between adenomatous polyps and cholesterol polyps by incorporating two independent risk factors: decreased miR-33b levels and an elevated miR-33a/miR-33b ratio. The model at the 0.424 optimal cut-off had an area under the curve (AUC) of 0.885, specificity of 84.62%, and sensitivity of 83.33%. In addition, the bootstrap internal validation through 1,000 iterations showed that the model had good performance (average AUC: 0.885, 95% CI: 0.752–1.000).

Our findings reveal that miR-33 plays a significant role in the formation and development of cholesterol polyps, highlighting its potential as a novel diagnostic biomarker for differentiating between cholesterol and adenomatous polyps. By identifying distinct miRNA profiles associated with different polyp types, this model provides clinicians with a powerful diagnostic tool to reduce diagnostic uncertainty, improve the accuracy of adenoma prediction, and optimize indications for cholecystectomy. This approach can significantly enhance clinical decision-making by minimizing missed diagnoses of adenomatous polyps, while also preventing unnecessary surgeries for benign cholesterol polyps.

Furthermore, the integration of miR-33-based diagnostics into clinical practice could lead to the development of more refined and evidence-based guidelines for gallbladder polyp management. By providing a molecular basis for differentiating polyp types, miR-33 could complement existing imaging criteria, enhancing the overall diagnostic framework. This could ultimately result in more personalized and precise treatment strategies, aligning with the broader trend toward precision medicine in clinical practice. For example, patients with cholesterol polyps, which are typically benign, could be managed conservatively with regular monitoring, thereby avoiding the risks and complications associated with cholecystectomy. On the other hand, patients with adenomatous polyps, which have a higher risk of malignancy, could be prioritized for surgical intervention. This targeted approach not only improves patient outcomes by reducing the incidence of unnecessary surgeries but also optimizes healthcare resource utilization.

Due to the low incidence of certain polyp subtypes and the strict inclusion criteria (requiring histopathologically confirmed polyp subtypes), our sample size was small. Nevertheless, it still met the Event Per Variable (EPV) requirements. According to the EPV principle proposed by Peduzzi et al. [56] in 1996, approximately 5–10 events per independent risk factor were needed to ensure sufficient model accuracy. In our study, this translated to requiring 10–20 patients with adenomatous polyps in the development cohort.

While our results are statistically significant and meet EPV standards, we admit that the small sample size may limit the statistical power and generalizability of the results. This limitation was primarily due to the possibility of sampling bias. With a small sample, it is more likely that the characteristics of the included subjects may not accurately represent the entire population of interest. This could lead to over-or under-estimation of miR-33’s performance in classifying the relevant conditions. We fully acknowledge that miR-33 cannot be readily implemented in clinical settings at this stage. Further validation in larger and more diverse cohorts is essential to confirm the clinical utility and generalizability of our findings. To this end, we have initiated collaborations with two additional large-scale medical centers, which are geographically diverse and possess extensive patient databases relevant to our study, enabling us to test our model on a wide range of patients with varying genetic backgrounds, environmental exposures, and healthcare access. Future studies should also evaluate the model’s performance in diverse patient populations and clinical settings to ensure broad applicability and reliability.

Besides, obtaining gallbladder mucosa samples for miR-33 analysis is invasive, which challenges its widespread clinical implementation and limits its use as a routine diagnostic tool in clinical practice. Future research should explore the diagnostic potential of serum miR-33 levels as a non-invasive alternative. Such an approach could facilitate preoperative polyp type identification, further enhancing the clinical utility of our findings.

## Conclusion

In conclusion, this study demonstrates the potential of miR-33 as a new diagnostic biomarker for differentiating between cholesterol and adenomatous polyps. These findings can assist clinicians in making more informed diagnostic and treatment decisions, ultimately enhancing patient care in the management of gallbladder polyps. Although our results are promising, the current model still needs further optimization and validation before it can be easily applied in clinical practice. Future research should give priority to multicenter collaborations and prospective designs to strengthen the validity of the findings and evaluate the model’s performance in real-world clinical settings. Exploring the interplay among lipid metabolism, miRNA expression, and gallbladder polyps could lead to new therapeutic strategies and ultimately lead to enhanced patient outcomes.

## Data Availability

The datasets used and/or analyzed during the current study are available from the corresponding author on reasonable request.
